# Sphingolipids: Less Enigmatic but Still Many Questions about the Role(s) of Ceramide in the Synthesis/Function of the Ganglioside Class of Glycosphingolipids

**DOI:** 10.3390/ijms25126312

**Published:** 2024-06-07

**Authors:** Cara-Lynne Schengrund

**Affiliations:** Department of Biochemistry and Molecular Biology, The Pennsylvania State University College of Medicine, Hershey, PA 17033, USA; cxs8@psu.edu

**Keywords:** ceramide, ceramide transport protein, ganglioside, glycosphingolipid, glycosyltransferases, sphingosine

## Abstract

While much has been learned about sphingolipids, originally named for their sphinx-like enigmatic properties, there are still many unanswered questions about the possible effect(s) of the composition of ceramide on the synthesis and/or behavior of a glycosphingolipid (GSL). Over time, studies of their ceramide component, the sphingoid base containing the lipid moiety of GSLs, were frequently distinct from those performed to ascertain the roles of the carbohydrate moieties. Due to the number of classes of GSLs that can be derived from ceramide, this review focuses on the possible role(s) of ceramide in the synthesis/function of just one GSL class, derived from glucosylceramide (Glc-Cer), namely sialylated ganglio derivatives, initially characterized and named gangliosides (GGs) due to their presence in ganglion cells. While much is known about their synthesis and function, much is still being learned. For example, it is only within the last 15–20 years or so that the mechanism by which the fatty acyl component of ceramide affected its transport to different sites in the Golgi, where it is used for the synthesis of Glu- or galactosyl-Cer (Gal-Cer) and more complex GSLs, was defined. Still to be fully addressed are questions such as (1) whether ceramide composition affects the transport of partially glycosylated GSLs to sites where their carbohydrate chain can be elongated or affects the activity of glycosyl transferases catalyzing that elongation; (2) what controls the differences seen in the ceramide composition of GGs that have identical carbohydrate compositions but vary in that of their ceramide and vice versa; (3) how alterations in ceramide composition affect the function of membrane GGs; and (4) how this knowledge might be applied to the development of therapies for treating diseases that correlate with abnormal expression of GGs. The availability of an updatable data bank of complete structures for individual classes of GSLs found in normal tissues as well as those associated with disease would facilitate research in this area.

## 1. Introduction

During the 140 years since glycosphingolipids (GSLs) were first named [1], they have been found to consist of a lipophilic moiety, ceramide, that can vary in composition and to express a wide variety of carbohydrate components (e.g., Ref. [2]). In cells, the polar carbohydrate portion is generally found on the outer surface of the plasma membrane, while the hydrocarbon chains of the ceramide portion interact with those of the phospholipids and cholesterol present in the lipid bilayer. As a result of the polar components of the GSLs interacting with each other and the Van der Walls attractions between their hydrocarbon chains and those of membrane lipids, GSL-enriched lipid microdomains (GEMs) [3,4], commonly referred to as lipid rafts [4], are formed. Regardless of how GEM domains are identified, it is known that GSLs can be found in clusters where they can function as “multivalent” ligands. This is important as individual carbohydrate moieties tend to be bound weakly, while a multivalent presentation of the same ligand can be bound more tightly by molecules expressing multiple binding sites for the carbohydrate ligand expressed [5]. As a result, GSLs can function, for example, as receptors in signal transduction [6] as well as binding sites for a variety of pathogens [7]. To understand how variations in either portion of a GSL affect its function, the combined contribution of each must be considered, for example, how ceramide affects downstream synthesis of the carbohydrate chain, the effectiveness of signal transduction initiated by binding a ligand to its GSL saccharide receptor, or the ability of a pathogen to interact with its target cell. 

The first portion of this review focuses on the synthesis of ceramide and the problems that can arise as a result of the disruption of specific steps, while the second part will discuss ceramide-containing GSLs of the ganglio (Gg) series. This split reflects the manner in which sialic acid-containing Gg sphingolipids, called gangliosides (GGs), were traditionally studied. With improvements in methodology, the question of the effects that changes in ceramide composition might have on the function of a GG is currently being investigated. The selection of just GGs for discussion reflects not only the number of GGs identified [8] but also the broad clinical interest in them due to their elevated concentration in the nervous system as well as their identification as cell markers for certain cancers. Because their synthesis is carried out by glycosyltransferases that can often act in the synthesis of more than one GG, two areas to investigate about the effect of the composition of ceramide on that process are whether it influences the transport of the partially synthesized GG or the activity of the enzyme. The scope of this review is limited to these two areas in order to highlight some of the gaps in what has been published about ceramide and ganglioside synthesis and how errors in one of those steps may contribute to disease. It is anticipated that this will lead readers to ask similar questions about steps in GSL catabolism.

## 2. Synthesis of Ceramide

**Figure 1:**
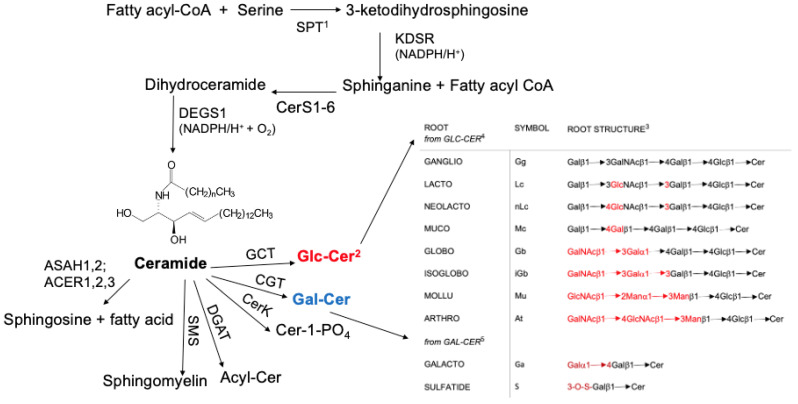
Overview of sphingolipid metabolism. **^1^** Enzyme abbreviations: SPT, serine palmitoyltransferase (EC 2.3.1.50); KDSR, 3-ketodehydrosphingosine reductase (EC 1.1.1.102); CerS1-6, ceramide synthases 1-6 (EC 2.3.1.299); DEGS1, dihydroceramide desaturase 1 (EC 1.14.19.17); ASAH1, acid ceramidase (EC 3.5.1.23); ASAH2, neutral ceramidase (EC 3.5.1.23); ACER1,2,3, alkaline ceramidases (EC 3.5.1.23); SMS, sphingomyelin synthase (EC 2.7.8.27); DGAT, diacylglycerol acyltransferase (EC 2.3.1.20); CerK, ceramide kinase (EC 2.7.1.138); CGT, ceramide galactosyltransferase (EC 2.4.1.47); and glucosyl ceramide transferase (UGCG, EC 2.4.1.80). **^2^** Bold red and blue emphasize initial GSL products produced from Cer. **^3^** In the list of products from Glcβ1- and Galβ1-1Cer, red indicates differences in saccharide composition. **^4^** Glcβ1^_^1Cer serves as the precursor for a number of different classes of GSLs. **^5^** Synthesis of Galβ1^_^1Cer provides a base component for the synthesis of just 3 different types of GSLs: formation of sulfatide (3-O-sulfogalactosylceramide, SM4), sialylation to form GM4, or addition of a galactose moiety to form Ga2. Long-chain hydroxylated fatty acids tend to be present in the ceramide components of these compounds [21,22].

The synthesis of ceramide (Figure 1) takes place in the endoplasmic reticulum and is initiated by the rate-limiting reaction of sphingolipid biosynthesis. This is catalyzed by serine palmitoyl transferase (SPT), which usually catalyzes the linkage of the fatty acid component of fatty acyl-CoA to the primary amine on serine. It should be noted that SPT is promiscuous and is known to use amino acids and fatty acyl-CoA derivatives other than serine and palmitoyl CoA [9]. 3-ketodihydro-sphingosine reductase (KDSR) then catalyzes its reduction to yield sphinganine [10], to which one of six ceramide synthases (CerSs) catalyzes the addition of a fatty acyl or an αOH fatty acyl residue from CoA [11]. The findings that CerSs are expressed in different organs (Table 1) help to explain why gangliosides having the same carbohydrate portion can vary in their ceramide components, which in turn may affect their function. Dihydroceramide desaturase (DEGS) then catalyzes the introduction of a C4-trans double bond into the sphinganine component to yield ceramide [12], which can serve as the substrate for a number of different enzymes, as depicted in Figure 1. For a complete summary, see [13]. Not shown and not discussed in this review is the fact that sphingosine kinase 1 (SphK1) can catalyze the conversion of sphingosine to sphingosine-1-phosphate (S1P), which is found to enhance angiogenesis [14]. In contrast, ceramide is antiangiogenic. In glioblastoma, S-1-P expression is about 9 times that found in normal gray matter, while ceramide is found at about 1/5 of that found in normal gray matter [14]. Inhibition of sphingosine kinase 1 (SphK1) in glioblastoma cells cocultured with endothelial cells blocked angiogenesis, supporting the question of whether an effective SphK1 inhibitor would be a treatment for glioblastoma [14]. The finding that the dysregulation of the expression of a number of enzymes needed for the synthesis of ceramide is associated with various diseases (Table 1) makes it important to understand the contribution of both the sphingosine base and the fatty acid components of ceramide to the formation of downstream GSLs known to exert both positive and negative clinical effects. For a review of the role of regulatory pathways in ceramide metabolism, see [15].

While there are a number of different series of glycosphingolipids (GSLs), whose synthesis is initiated with Glc-Cer, this review focuses on the ganglio (Gg) series. It should be noted that the only compound referred to as a ganglioside that is synthesized from Gal-Cer is GM4, which is actually a galacto-GSL (Figure 1). Support for looking specifically at whether ceramide composition affects the synthesis/function of members of a specific class of GSLs is provided by findings such as (1) a close correlation between the fatty acyl component of ceramide and the terminal carbohydrate composition of the GSL [16]; (2) the sphingosine composition differed in specific gangliosides isolated from brains of animals [17]; (3) GT1c, containing ceramide (d18:1/24:1), might be a marker for glioblastoma multiforme [18]; (4) lipid components of ceramide affect its interaction with membrane lipids and proteins [19]; and (5) GGs with a shorter fatty acyl chain have a much higher immunosuppressive activity than those with longer fatty acyl residues [20].

**Table 1:** Proteins involved in the synthesis of galactosyl and glucosyl ceramide from a fatty acyl- CoA, an amino acid (usually serine), and either UDP-galactose or UDP-glucose.

Enzyme/ Transfer Protein ^1^	Substrate Specificity	Co-Factors	Tissue Distribution	Subcellular Distribution	Examples of Diseases Due to Altered Protein Activity
SPT1,2 SPT1,3	Predominantly C16-CoA + serine C14-C18 acyl COAs [23]	Pyridoxal 5′-phosphate [24]	Proliferating tissue [25]	ER [26]	HSN1 ^2^ [27] ALS [28]
KDSR	3-ketodihydro-sphingosine	NADPH/H^+^	Broad [29]	ER [30]	Maintenance of leukemia cell survival [31]
CerS1 ^3^	C18-CoA + sphin- ganine/sphingosine [32]		Brain (primarily neurons [33], skeletal muscle, testis [32]	ER	Head and neck squamous cell carcinoma [34]
CerS2	C20-26-CoA + sphinganine/sphingosine [32]		Kidneys, liver, spleen, intestine, bone marrow, lymph nodes, and thymus [32]	ER	Defective myelination, followed by neurological decline [35]
CerS3	C22-C26-CoA + sphinganine/sphingosine [32]		Primarily testis, skin, and prostate [32]	ER	Congenital ichthyosis [36] Used commercially to treat psoriasis [37]
CerS4	C18-C20-CoA + sphinganine/sphingosine [32]		Broad, with more in heart, leukocytes, skin, and spleen	ER	KRAS-mutant colorectal cancer [38]
CerS5	C16-CoA + sphinganine/sphingosine [39]		Low but more in prostate and skeletal muscle	ER	Mice more susceptible to inducible colitis and associated colon cancer [40]
CerS6	C14-C16-CoA + sphinganine/sphingosine [32]		Low but it is in intestine, spleen, thymus, and lymph nodes	ER	Overexpression in acute lymphoblastic leukemia enhances resistance to chemotherapy [41] as well as triple-negative breast cancer [42]
DEGS1	Dihyroceramide	NADPH/H^+^	Ubiquitous but greater in liver, Harderian gland, kineys, and lungs [43]	ER/mitochondria [44]	Decreased activity associated with severe neurological defects [45]
CERT	Ceramide with C14-C20 fatty acids [46]	ATP-dependent [47]	Ubiquitous [48]	ER + Golgi Cytosol [47]	Affects diabetes [49], Alzheimer’s senescence [50] Developmental disorders [51]
ASAH1	Ceramide, preferably with C16:0-C18:0 [52]	Saposin D [53]	Ubiquitous, especially in heart and kidneys [54]	Lysosomes [54]	LSD, Farber’s disease, spinal muscular atrophy [55]
ASAH2 (nCDase)	Preferably, ceramides + dihydroceramides [56]		Strongest expression is in small intestine + colon [57]	Plasma membrane, Golgi, mitochondria	Highly related to progression of colon cancer [58]
ACER1	Preferably, ceramides with VLFAs C24:0, C24:1 [59]	Ca^2+^	Predominantly in skin cells + hair follicle stem cells [54]	ER + Golgi [54]	Decrease leads to alopecia [54]
ACER2	Prefers C18:1-C20:1, C20:4 ceramides [60] + dihydro- ceramides [56]	Ca^2+^	Intestines [54] + placenta [60], less so in other tissues	Golgi + ER [54]	Regulation of protein glycosylation in the Golgi complex [60]
ACER3	Ceramides, dihydroceramides + phytoceramides with C18:1, C20:1 acyl chains [61]	Ca^2+^	Ubiquitous	Golgi + ER [54]	Deficiency leads to progressive leukodystrophy [62], colitis and its associated tumorigenesis [63]
SMS1 SMS2	Ceramide and phosphatidyl-choline [64]		Ubiquitous [65]	1—Golgi [66]; 2—plasma mebrane [67]	SMS1 is necessary for cell survival SMS2 deficiency less severe (for a review, see [64])
DGAT2	Ceramide, diacylglycerol	Long-chain acyl-CoA synthase 5 [68]	Ubiquitous	Domains of ER near mitochondria-associated membranes [69]	Essential for mice to survive [66]
CERK	D-*erythro*-ceramide with an acyl chain ≥ C12 and trans C=C in sphingosine [70]	Ca^2+^	Neutrofils, cerebellar granule cells, epithelial-derived lung carcinoma cells [70]	*trans*-Golgi [71]	Cer1P enhances inflammatory response [72]
CGT	Ceramide with 2-OH [73] and long-chain fatty acids [74] + UDP-Gal	Sig-1R negatively regulates CGT activity [75]	Oligodendrocytes, Schwann cells, kidneys, and testes [76]	ER and nuclear membrane [77]	Defective myelination [21] Receptor for HIV-1 [78] Krabbe disease if turnover is inhibited [79]
GCT	Ceramide with usually C16, C24 fatty acids [19] + UDP-Glc	RTN1-C promotes activity	Broad	Golgi	Venous malformation [80] Gaucher’s disease [81]

**^1^** The only protein abbreviation not included in the footnote for Figure 1 is CERT: ceramide transfer protein. **^2^** HSN1, human hereditary sensory and autonomic neuropathy 1; ALS, amyotrophic lateral sclerosis; Sig-1R, sigma-1 receptor; HIV-1, human immunodeficiency virus-1. **^3^** Ceramide synthases act on both sphingosine and sphinganine [82]. It can be seen that even a change in activity of one of the ceramide synthases can induce a change in ceramide product associated with a specific clinical problem. For example, failure to synthesize Gal-Cer, needed by oligodendroglia, can result in defective myelination [35].

## 3. Synthesis of Glc- and Gal-Cer

The proteins needed for the synthesis of ceramide, its transport from the ER to the Golgi, and the synthesis of Glc- and Gal-Cer are shown in Table 1, as are examples of possible clinical problems that alterations in the activity of a protein needed for their synthesis might cause. When considering potential problems, care should be taken to look at the possible effects that changes in the synthesis of specific ceramides may cause directly or have on the synthesis of downstream GSLs, which have been implicated in disease. Because of the diversity of functions identified for GSLs, it is imperative to understand the effect different ceramide compositions might have not only on proteins needed for the metabolism of GSLs but on their function as well. 

The results of studies of the possible effects that sphingosine moieties having different chain lengths (C12-C20) and a constant fatty acyl group might have on membranes showed that those with chains shorter than C16 did not enhance gel-phase formation. This was postulated to result from the weakening of the Van der Waals attractions between hydrocarbon chains [83]. Sphingosine length has also been reported to affect the fluctuation and extension of the GG headgroup above the membrane surface [84]. Studies of the effect of different fatty acyl components on the interaction of ceramide with membranes have also provided information about how they might affect membrane function. For example, studies of the effect(s) of saturated and unsaturated fatty acyl groups on membrane order indicated that saturated fatty acyl moieties in ceramide increased order and enhanced gel/fluid separation, while unsaturated fatty acyl moieties having the same chain length had either less, when it was C24:1, or no, in the case of C18:1, ability to form gel domains at 37 °C [85]. These observations appear to correlate with the melting points of the fatty acid components per se, which are known to increase with the hydrocarbon chain length of saturated ones due to Van der Waals forces between the chains, allowing them to pack more tightly than those with shorter ones [86]. The introduction of cis-double bonds disrupts that packing and correlates with a reduction in the melting point (e.g., C18:0 at 69 °C; C18:1^D9^ at 13 °C). The ability of very-long-chain ceramide components to interdigitate allows them to form tubular structures [85]. Combined, it can be seen that variations in the composition of ceramide could have a marked effect on the ability of gangliosides with the same carbohydrate moiety to exert the same cellular functions. An example of why this might be clinically important is the finding that the fatty acid composition of ceramide found in the GGs GM3 and GD3 isolated from bovine and mature human milk differed significantly, with bovine milk having more long-chain (≥C20:0) fatty acids than human milk [87]. Of particular interest was the finding that tricosanoic acid (C23:0) accounted for about 25% of the fatty acid content of GM3 and GD3 isolated from bovine milk, while it was about 10-fold lower in GM3 and GD3 isolated from human milk [87]. The repetition of these studies using a different approach (low-energy collision-induced dissociation (CID) measurements and tandem mass spectrometry obtained at stepped higher-energy CID) also identified tricosanoic acid as a major fatty acid component of GM3 from bovine milk [88]. Differences in the fatty acid component could affect the interaction of the gangliosides with cells and, if they become components of the cell membrane, their function in lipid rafts. Interestingly, the amount of GD3 in human milk decreases with time from birth [89], permitting one to ask whether there were changes in ceramide over that period. An obvious question these observations raise is whether the fatty acid differences between GM3 and GD3 in cow and mature human milk affect infant digestion/nutrition or if there are other differences in the composition of bovine milk that make it less suitable than human milk for human newborns. These observations support exploring what effect ceramide composition may have on the metabolism and function of GSLs and, more specifically, in this review, on GGs.

## 4. Role of Ceramide Composition in Sphingolipid Transport

The identification of different sites of GSL synthesis within the Golgi points out the need for transport of Glc- and Gal-Cer from their original sites of synthesis to sites of modification. The identification of transporters and their characterization have provided some answers about the role ceramide composition may have. 

Evidence indicates that the ceramide transport protein (CERT) carries ceramide from the cytosolic side of the ER to the trans-Golgi, where it can be galactosylated, while vesicles are proposed to carry ceramide from the cytosolic side of the ER to the cis-Golgi, where it is glucosylated [90] (for a review, see [91]). Studies of CERT specificity for ceramides having different acyl groups indicated that those having a long-chain α-hydroxy fatty acid were transported by CERT more efficiently than those with non-hydroxylated ones [11]. Additional support for these observations is provided by the findings that α-OH fatty acids are found in Gal-Cer-containing GSLs such as sulfatide [92] and GM4 [93]. After the conversion of Cer transported to the cis-Golgi to Glc-Cer, it is carried by a member of the glycolipid transport protein (GLTP) family [94] to the trans-Golgi. Justification for the hypothesis that the altered ceramide composition could affect the use of Glc-Cer is provided by observations that show that GLTP transported Glc-Cer with shorter acyl chains (C8,12,16) to the trans-Golgi for additional glycosylation more effectively than it did those with longer acyl chains (C22:0 and C24:1, or C24:0) which had the poorest transport [95]. This was supported by an interesting use of surface plasmon resonance (SPR) to measure the rate of GLTP removal of Glc-Cer having specific fatty acyl moieties in its ceramide component from an SPR sensor chip [95]. GLTP has also been found to mediate the transport of the GG, GM1, in a non-vesicular manner between “native” membranes [96] and to act as a regulator of GSL levels in cells [97]. Structural studies have shown that it is the fold present in the GLTP structure that is responsible for binding the ceramide component of the sphingolipid, thereby contributing to the specificity of the sphingolipids bound [95,98,99]. Combined, these observations support further exploration of whether ceramide composition affects not only the transport of GSL precursors but also the activity of glycosyl transferases and glycosidases. As a result of the number of different GSLs identified, this portion of the review focuses on the roles of ceramide in the synthesis/function of the oligosaccharide portion of gangliosides (GGs), reflecting this reviewer’s interest in their neural function(s).

## 5. Why Focus on the Effect of Ceramide Composition on Gangliosides

**Figure 2:**
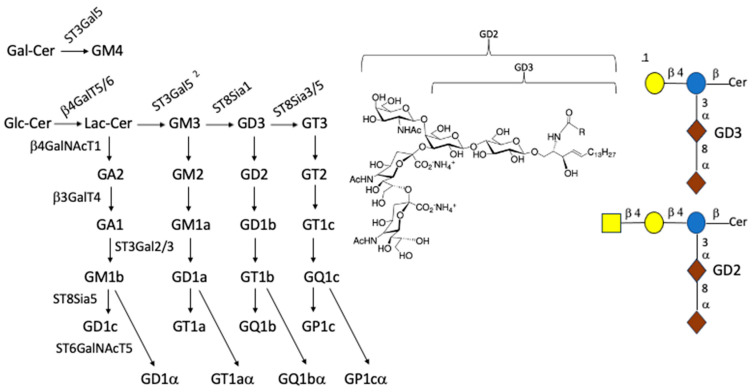
Synthesis of the oligosaccharide portion of gangliosides. **^1^** Symbols used to indicate sugars of GD3 and GD2 are the following: blue circles = Glc; yellow circles = Gal; yellow squares = N-acetylgalactosamine; and deep red = sialic acid [122]. Definitions for the gangliosides are given in the legend for Figure 2, with the exception of α, which indicates presence of sialic acid-linked α2-6 to N-acetylgalactosamine. **^2^** Enzyme abbreviations are the following: UGCG, UDP-glucose:ceramide β1-1′glucosyltransferase; ST3Gal5, ST3 β-galactoside α-2,3-sialyltransferase; β4GalT5/6, UDP-galactose: glucosylceramide β1-4 galactosyl transferase (lactosylceramide synthase) [123,124]; β4GalNT1, UDP-GalNAc: LacCer/GM3/GD3/GT3 β1-4 N-acetylgalactoseaminyl transferase (ganglioside GA2, GM2, GD2, and GT2 synthase); B3GalT4, UDP-galactose: GA2/GM2/GD2/GT2 β1–3 galactosyl transferase (ganglioside GA1, GM1a, GD1b, and GT1c synthase); ST3Gal5, CMP-sialic acid: lactosyl-ceramide α2-3 sialyltransferase (GM3 synthase); ST8SIA1, CMP-sialic acid: GM3 α-2,8-sialyltransferase (GD3 synthase); ST8SIA3/5, CMP-sialic acid: GD3 α-2,8-sialyltransferase (GT3 synthase); ST8Sia5, CMP-sialic acid: GM1b α-2,8-sialyltransferase (GD1c synthase); and ST6GalNT5, ST6 N-acetylgalactosaminide α-2,6-sialyltransferase (mediates breast cancer metastasis to the brain [104]).

While each class of GSLs merits study, the Gg series of GSLs containing sialic acid, known as GGs, are of particular interest due to the expression of their sialic acid-containing carbohydrate residues on the outer surface of the cell’s plasma membrane, with their carbohydrate head group extending outward as part of the glycocalyx [100] and their presence in lipid rafts [101]. As a whole, they can affect such processes as neural development and function [19,102,103], cell growth and metastasis [104], angiogenesis [105], and immunosuppression [106,107] via interaction with external ligands and their varied effects on signal transduction. See Table 2 for the carbohydrate compositions and Figure 2 for a schematic representation of steps in the synthesis of the gangliosides discussed. They provide a sample of the more than 200 different carbohydrate compositions identified for GGs thus far [8]. The multiplicity of products formed from a single GSL precursor is evidenced by the ability of Lac-Cer to serve as the precursor for a number of GSL series in addition to Ggs, including those in the lacto, neolacto, muco, globo, and isoglobo series (Figure 1). This raises the question of whether any of the glycosyl transferases catalyzing the addition of sugar moieties to Lac-Cer is influenced by the composition of the ceramide component.

**Table 2:** Carbohydrate composition of some common gangliosides.

Name ^1^	Carbohydrate Composition ^2^
Lac-Cer	Gal(ß1–4)Glcß1–
GM3	SA(α2–3)Gal(ß1–4)Glcß1–
GM2	GalNAc(ß1–4)[SA(α2–3)]Gal(ß1–4)Glcß1–
GM1a	Gal(ß1–3)GalNAc(ß1–4)[SA(α2–3)]Gal(ß1–4)Glcß1–
GA2	GalNAc(ß1–4)[SA(α2–3)]Gal(ß1–4)Glcß1–
GA1	Gal(ß1–3)GalNAc(ß1–4)Gal(ß1–4)Glcß1–
GM1b	SA(α2–3)Gal(ß1–3)GalNAc(ß1–4)Gal(ß1–4)Glcß1–
GD1a	SA(α2–3)Gal(ß1–3)GalNAc(ß1–4)[SA(α2–3)]Gal(ß1–4)Glcß1–
GT1a	SA(α2–8)SA(α2–3)Gal(ß1–3)GalNAc(ß1–4)[SA(α2–3)]Gal(ß1–4)Glcß1–
GT1b	SA(α2–3)Gal(ß1–3)GalNAc(ß1–4)[SA(α2–8)SA(α2–3)]Gal(ß1–4)Glcß1–
GQ1b	SA(α2–8)SA(α2–3)Gal(ß1–3)GalNAc(ß1–4)[SA(α2–8)SA(α2–3)]Gal(ß1–4)Glcß1–

**^1^** The nomenclature used is basically that of Svennerholm [108], in which G stands for ganglioside; the following capitalized letter refers to the number of sialic acid residues (A = 0, M = 1, D = 2, T = 3, Q = 4), and the Arabic numeral (e.g., 1, 2, etc.) refers to the number of sugars in the GG backbone based on their mobility upon thin-layer chromatography. In this system, the number of sugars in the backbone can also be obtained by subtracting the Arabic numeral from five. Lower-case letters indicate the placement of sialic acid residues on the internal galactosyl residue, as follows: a indicates one; b, two, except in the case of GM1b, where it is on the external galactose; c, three, etc. SA indicates sialic acid, the name used to indicate the presence of a neuraminic acid derivative [109]. **^2^** In each case, Glc is β1-1-linked to ceramide.

In terms of neuronal behavior, gangliosides are found in the highest concentration in the brain [110], which contributes to our interest in them. Their composition changes during neural development [102,111], and there is a close association between errors in their metabolism, both anabolism and catabolism, and numerous clinical problems (e.g., Refs. [112,113,114]. Note that a glycosyl transferase is often promiscuous, recognizing multiple GG substrates. Hence, changes in its activity can affect the synthesis of multiple products, possibly multiplying the effect. The results from a number of different studies in which the expression of a protein needed to synthesize a GG or GGs was knocked out indicated that they are essential for normal functions such as cell growth [115,116], neuronal maturation [102,111], and neuronal transmission [117]. Examples of human clinical problems related to defects in neuronal function include infantile-onset symptomatic epilepsy (failure to synthesize GM3 [113]), Tay–Sach’s disease (failure to degrade GM2 [114,118]), Parkinson’s (lack of GM1 [103]), and Alzheimer’s disease [119,120]. More generally, gangliosides can affect angiogenesis [105,121] and metastasis [104,116]. The identification of the cause of infantile-onset symptomatic epilepsy in humans [113] confirmed the need for gangliosides for normal neural development in people.

## 6. Contribution of Glycan and Ceramide to GG Function

Studies of the glycan components of GSLs have shown that the glycan component is the epitope that can interact with components either on the surface of or external to the cell, while the composition of the ceramide portion located within the cell membrane can affect their possible location and interactions between membrane components [84]. This supports the hypothesis that the ceramide component of GSLs may affect signal transduction initiated by epitope interactions. Table 3 includes examples of phenotypes induced by the altered expression of specific GGs for which alterations in ceramide composition are given when known. The following provides an example of the role of the ceramide portion of GM3 in type 2 diabetes and metabolic disease [125]. 

Increased concentrations of GM3 were found to downregulate insulin receptor activity in 3T3-L1 adipocytes by causing the insulin receptor to move out of the caveolae to other areas of the membrane, and when it was decreased, the receptor was seen to move back into the caveolae, thereby restoring receptor function [126]. The results of studies of mice unable to synthesize ceramide-containing C22-C24 fatty acyl residues showed that insulin receptors in the liver were unable to move back into the caveolae and remained inactive. Studies of serum lipoproteins indicated that GM3 is significantly elevated in severely obese patients with type 2 diabetes and in atherosclerotic lesions. Analyses of the ceramide composition of the GM3 indicated that of the eight elevated species found in individuals with visceral fat accumulation and metabolic disease, all had d18:1 sphingosine and six had hydroxylated fatty acyl groups, with h24:0 and h24:1 being the predominant types. This led to the proposal that GM3 (d18:1-h24:1) be used for metabolic screening [127]. For cells to have the very-long-chain fatty acids identified in the ceramide components of GM3, cellular very-long-chain fatty acid (VLCFA) elongases (ELOVL1-7) had to catalyze the elongation of the typical fatty acids synthesized [128]. In the early stages of metabolic syndrome, there is an increase in proinflammatory VLCFAs (22:0, 23:0, 24:0, h24:0) in serum and adipose tissue [129]. In severe obesity and metabolic syndrome, macrophage expression of VLCFAs decreases due to steroid regulatory element-binding protein SREBP1-induced reprograming of lipid synthesis [130]. The fact that the chain length of the sphingoid base may vary from 12 to 20 carbons [131] indicates that it, as well as the fatty acyl moiety, may affect the interaction of the ceramide moiety with cell membrane components. Combined, these results support a role for GM3, moderated by its ceramide composition, in diabetes and metabolic syndrome. While the exact effect of the chain length of the sphingosine component of ceramide on GSL function is still being studied, over 30 years ago it was found that gangliosides from sensory nerves had more d18:1 than those from motor nerves, which had more d20:1 [132]. The effect of this change on GG function remains unknown.

**Table 3:** Examples of some GGs associated with clinical phenotypes.

Ganglioside	Site	Fatty Acid	Sphingoid Base	Clinical Functions Affected by Altered GG Expression
Lac-Cer	Neutrophils [106] Human milk [133]	Primarily C24:0,24:1, shorter chains not effective [106], predominantly ≥ C20 [133]	d18:1 > d20:1 in human milk [133]	Outside-in signaling [106,134]; skin problems, atherosclerosis, mitochondrial disfunction [135]
GM3	H^1^: adipose, muscle, liver + serum [127,129]; mature outer retina [136]	Primarily C18,22,24,24:1^w9^ [137]	d18:1 [137,138] d20:1 predominant in pyramidal and granular layers of the dentate gyrus [139]	Type 2 diabetes, metabolic disease, and innate immune response [127,128,129]
GD3	Prevalent in neuronal stem cells [140] Plasma membrane cytoplasm, ER-mitochondria-associated membranes [141]	Longer-chain fatty acids > C18:0 in adult human brain [142]	d18:1 > d20:1	Enhances proliferation via its roles in signal transduction (for a review, see [143])
GM1a	Abundant in mammalian brains enriched in white matter [144] and intestines [145] Plasma membrane and nucleus [102]	C16:1 [146] ≥ C14 [147]		C16:1 acyl inhibits GM1 clustering and reduces binding of certain toxins by affecting nanodomain structure [146] Sorting to lysosome [147] Lack of GM1a contributes to peripheral symptoms of Parkinson’s disease [148] Overexpression inhibits cell growth signals [149]
GM1 * (rat brain): GM1 in brain was analyzed as a single GSL [139]	Enriched in hippocampus and substantia nigra Corpus callosum	C18:0	d18:1 > d20:1 in hippocampus in Alzheimer’s disease (AD); d20:1 > d18:1 in controls [150,151]	Areas of the brain enriched with GM1 [139] It is hypothesized to facilitate Aβ assembly in AD [119,152]
GD1a	Enriched in brain in certain brain nuclei and tracts [144]			Age-related decline in peripheral tissues contributes to non-CNS symptoms of Parkinson’s disease [148]
GD1 * [139]	Hippocampus, cortex, periaqueductal gray area, interpeduncular nucleus, and substantia nigra, cortex, superficial gray superior colliculus	C18:1	Only d18:1 is in the middle molecular layer of the dentate gyrus, while only d20:1 is in the outer and inner layers [139]	Areas of the brain enriched with GD1 [139]
GD1b	Expressed in brain gray and white matter [144]			May function in axon–myelin stabilization as anti-GD1b correlated with abnormal myelin [153]
GT1b	Expressed in brain gray and white matter [144]			Contributes to nerve injury-induced spinal cord microglia activation and pain hypersensitivity [154]
GQ1b	Hippocampus	C18:0	D18:1	Temporal lobe epilepsy ^1^ [155]

* Indicates analysis of all GM1 or GD1 gangliosides. **^1^** Authors indicate that more samples need to be studied, as the results are from a single sample of an affected hippocampus and a single control hippocampus [155].

Despite the proliferation of studies concerning the roles of gangliosides in neural function (930 results on PubMed between 1969 and 6 April 2024), it can be seen in Table 3 that there are still numerous questions about their ceramide composition as well as the effect(s) it may have on function that are just starting to be explored, for example, the following in AD: What is the signal(s) that induces the synthesis of GM1 ceramide having less d20:1 sphingosine than that found in GM1 from the controls [150,151], and does it help facilitate Aβ assembly?; what is the ceramide composition of GGs whose expression correlates with specific disease states?; and, once known, how do these changes alter the role of the GG in signal transmission and/or cell survival? Answers to these questions may enable the more effective development of therapeutic agents for use in the treatment of diseases than those found in earlier attempts (e.g., for AD and Parkinson’s [156,157]; for a review, see [158]).

The above questions also apply to studies of the role(s) of GGs in cancer. Numerous studies have shown that cells from different types of cancer may overexpress a specific ganglioside that is frequently not found to any extent in normal cells and can often be used as a diagnostic marker [159]. In these instances, the ganglioside expressed may disrupt signal transduction pathways, thereby affecting the growth of cancer cells and metastatic ability (for a review, see [143]). Interestingly, gangliosides can be shed into the circulation [160], where they may also affect the immune response [161], making the cancer more refractory to treatment. As more is learned about the roles of tumor-associated gangliosides, research has led to studies of how to use them in treatment. For example, the identification of the disialoganglioside GD2 as a tumor cell marker for neuroblastomas (NBs) [162] and, to a somewhat lesser degree, for melanomas and retinoblastomas [163] led to studies of the use of anti-GD2 antibodies to treat NB. Data from clinical trials resulted in its approval by the USA Food and Drug Administration (Dinutuximab) and the European Medicines Agency (Dinutuximab beta) for treatment of cases of NB refractory to more conventional forms of therapy [164]. The results from follow-up studies of the effectiveness of Dinutuximab as a therapeutic for stage 4 NB patients [165] indicated that, while not always effective, it did improve both 5-year event-free survival as well as overall survival [166]. Currently, its use for the treatment of other cancers with GD2 as a marker is being studied [163]. Because GD2 is not expressed to any extent by normal cells, the antibody can be used to target therapeutic drugs to malignant cells with minimal effect on the brain, as the blood–brain barrier inhibits access [167]. 

## 7. Summary and Future Directions

Based on the foregoing and as stated in the abstract, there are still many questions about the possible roles of ceramide in the function of GGs, as well as how not just changes in ceramide composition but also those in the glycan composition can affect GG function. While the discussion in this review focuses on GGs, the information presented points out the need to look at both the lipid and saccharide components when studying GSLs, an approach investigators are starting to embrace. Looking at Table 3, it can be seen that there is a need for more analyses of the ceramide composition of specific GGs associated with disease. This information is necessary as evidence has shown that a change in just a single double bond (16:0 vs. 16:1) of a GG can cause different effects on nanodomain formation and function [146]. As pointed out by one researcher and cited in a footnote to Table 3, analyses of multiple GG samples from both controls and affected individuals are needed so that the significance of possible differences due to alterations in GG ceramide composition can be evaluated [155]. Recent advancements in technology to identify GG ceramide composition—through improvements in both the application of column chromatography to separate gangliosides by sialic acid class (M, D, T, etc.) and total carbon chain length of their ceramide component [168] as well as improvements in the use of mass spectrometry to identify ceramide composition (e.g., Refs. [16,85,155,169,170])—are allowing investigators to start to obtain this information. Another approach to defining the effect of alterations in the fatty acid component of ceramide was to look at the effect of substituting the fatty acyl component of the GG on its integration into a lipid bilayer [171]. Recently, a method was developed using a highly modular chemoenzymatic cascade assembly (MOCECA) strategy [172] that permits the synthesis of structurally relevant gangliosides in large-scale concentrations sufficient for large-scale study. Combined, the two approaches should provide researchers with the ability to obtain answers about how specific changes in the fatty acid component affect GG cellular function. It is anticipated that, in addition to studies already published (e.g., Refs. [146,147]) about the effect of ceramide composition on the packing of a GG in the membrane as well as on the surface exposure of its polar head group, research using GGs with appropriately defined ceramide components will help further our mechanistic understanding. All of this information is crucial to understanding the effects seen when specific changes in ganglioside composition are identified as causative agents in disease and for the development of useful therapies.

As this reviewer was looking at all of the work that has been carried out in this area, it became apparent that what would be helpful to all investigators in this field is the development of a GG (or general GSL) data bank freely available to all. It should be able to be constantly updated as new information is gleaned and include items such as GSL identity; saccharide composition; ceramide composition; tissue(s) expressing it; animal source; method(s) used to isolate/characterize the GSL; characterization of function; whether it is associated with a disease; protein activity it may affect; and researcher and contact information or publication. I put this out for someone interested and knowledgeable in computer programming to develop and make available. 

## Data Availability

Data presented in is available in the references cited.

## References

[1] Thudichum J.L.W. (1884). A Treatise on the Chemical Constitution of the Brain: Based throughout upon Original Researches.

[2] Klenk E. (1942). Über Die Ganglioside, Eine Neue Gruppe von Zuckerhaltigen Gehirnlipoiden. Biol. Chem..

[3] Kojima N., Hakomori S. (1991). Synergistic Effect of Two Cell Recognition Systems: Glycosphingolipid-Glycosphingolipid Interaction and Integrin Receptor Interaction with Pericellular Matrix Protein. Glycobiology.

[4] Simons K., Ikonen E. (1997). Functional Rafts in Cell Membranes. Nature.

[5] Lee R.T., Lee Y.C. (2000). Affinity Enhancement by Multivalent Lectin–Carbohydrate Interaction. Glycoconj. J..

[6] Sasaki N., Toyoda M., Ishiwata T. (2021). Gangliosides as Signaling Regulators in Cancer. Int. J. Mol. Sci..

[7] Schengrund C.-L., Schengrund C.-L., Yu R.K. (2023). Carbohydrates: Binding Sites and Potential Drug Targets for Neural-Affecting Pathogens. Glycobiology of the Nervous System.

[8] Sipione S., Monyror J., Galleguillos D., Steinberg N., Kadam V. (2020). Gangliosides in the Brain: Physiology, Pathophysiology and Therapeutic Applications. Front. Neurosci..

[9] Muthusamy T., Cordes T., Handzlik M.K., You L., Lim E.W., Gengatharan J., Pinto A.F., Badur M.G., Kolar M.J., Wallace M. (2020). Serine Restriction Alters Sphingolipid Diversity to Constrain Tumour Growth. Nature.

[10] Stoffel W., Lekim D., Sticht G. (1968). Metabolism of Sphingosine Bases, VIII. Distribution, Isolation and Properties of D-3-Oxosphinganine Reductase. Stereospecificity of the NADPH-Dependent Reduction Reaction of 3-Oxodihydrosphingosine(2-Amino-1-Hydroxyoctadecane-3-One). Biol. Chem..

[11] Mizutani Y., Kihara A., Chiba H., Tojo H., Igarashi Y. (2008). 2-Hydroxy-Ceramide Synthesis by Ceramide Synthase Family: Enzymatic Basis for the Preference of FA Chain Length. J. Lipid Res..

[12] Ternes P., Franke S., Zähringer U., Sperling P., Heinz E. (2002). Identification and Characterization of a Sphingolipid Δ4-Desaturase Family. J. Biol. Chem..

[13] Hannun Y.A., Obeid L.M. (2011). Many Ceramides. J. Biol. Chem..

[14] Abuhusain H.J., Matin A., Qiao Q., Shen H., Kain N., Day B.W., Stringer B.W., Daniels B., Laaksonen M.A., Teo C. (2013). A Metabolic Shift Favoring Sphingosine 1-Phosphate at the Expense of Ceramide Controls Glioblastoma Angiogenesis. J. Biol. Chem..

[15] Chaurasia B., Summers S.A. (2021). Ceramides in Metabolism: Key Lipotoxic Players. Annu. Rev. Physiol..

[16] Kannagi R., Nudelman E., Hakomori S. (1982). Possible Role of Ceramide in Defining Structure and Function of Membrane Glycolipids. Proc. Natl. Acad. Sci. USA.

[17] Schengrund C.-L., Garrigan O.W. (1969). A Comparative Study of Gangliosides from the Brains of Various Species. Lipids.

[18] Sarbu M., Petrica L., Clemmer D.E., Vukelić Ž., Zamfir A.D. (2021). Gangliosides of Human Glioblastoma Multiforme: A Comprehensive Mapping and Structural Analysis by Ion Mobility Tandem Mass Spectrometry. J. Am. Soc. Mass Spectrom..

[19] Mencarelli C., Martinez–Martinez P. (2013). Ceramide Function in the Brain: When a Slight Tilt Is Enough. Cell. Mol. Life Sci..

[20] Ladisch S., Li R., Olson E. (1994). Ceramide Structure Predicts Tumor Ganglioside Immunosuppressive Activity. Proc. Natl. Acad. Sci. USA.

[21] Reza S., Ugorski M., Suchański J. (2021). Glucosylceramide and Galactosylceramide, Small Glycosphingolipids with Significant Impact on Health and Disease. Glycobiology.

[22] Takahashi T., Suzuki T. (2012). Role of Sulfatide in Normal and Pathological Cells and Tissues. J. Lipid Res..

[23] Lone M.A., Hülsmeier A.J., Saied E.M., Karsai G., Arenz C., von Eckardstein A., Hornemann T. (2020). Subunit Composition of the Mammalian Serine-Palmitoyltransferase Defines the Spectrum of Straight and Methyl-Branched Long-Chain Bases. Proc. Natl. Acad. Sci. USA.

[24] Yard B.A., Carter L.G., Johnson K.A., Overton I.M., Dorward M., Liu H., McMahon S.A., Oke M., Puech D., Barton G.J. (2007). The Structure of Serine Palmitoyltransferase; Gateway to Sphingolipid Biosynthesis. J. Mol. Biol..

[25] Batheja A.D., Uhlinger D.J., Carton J.M., Ho G., D’Andrea M.R. (2003). Characterization of Serine Palmitoyltransferase in Normal Human Tissues. J. Histochem. Cytochem..

[26] Han G., Gupta S.D., Gable K., Niranjanakumari S., Moitra P., Eichler F., Brown R.H., Harmon J.M., Dunn T.M. (2009). Identification of Small Subunits of Mammalian Serine Palmitoyltransferase That Confer Distinct Acyl-CoA Substrate Specificities. Proc. Natl. Acad. Sci. USA.

[27] Dawkins J.L., Hulme D.J., Brahmbhatt S.B., Auer-Grumbach M., Nicholson G.A. (2001). Mutations in SPTLC1, Encoding Serine Palmitoyltransferase, Long Chain Base Subunit-1, Cause Hereditary Sensory Neuropathy Type I. Nat. Genet..

[28] Dunn-Giroux T., Gable K., Gupta S.D., Mohassel P., Nalls M., Donkervoort S., Piccus Z., Majumder S., Proia R.L., Le Pichon C.E. (2020). SPTLC1 Mutations Associated with Early Onset Amyotrophic Lateral Sclerosis. FASEB J..

[29] (2024). The Human Protein Atlas Feb. https://www.proteinatlas.org/ENSG00000119537-KDSR/tissue.

[30] Spears M.E., Lee N., Hwang S., Park S.J., Carlisle A.E., Li R., Doshi M.B., Armando A.M., Gao J., Simin K. (2022). De Novo Sphingolipid Biosynthesis Necessitates Detoxification in Cancer Cells. Cell Rep..

[31] Liu Q., Chan A.K.N., Chang W.H., Yang L., Pokharel S.P., Miyashita K., Mattson N., Xu X., Li M., Lu W. (2022). 3-Ketodihydrosphingosine Reductase Maintains ER Homeostasis and Unfolded Protein Response in Leukemia. Leukemia.

[32] Levy M., Futerman A.H. (2020). Mammalian Ceramide Synthases. IUBMB Life.

[33] Becker I., Wang-Eckhardt L., Yaghootfam A., Gieselmann V., Eckhardt M. (2008). Differential Expression of (Dihydro)Ceramide Synthases in Mouse Brain: Oligodendrocyte-Specific Expression of CerS2/Lass2. Histochem. Cell Biol..

[34] Karahatay S., Thomas K., Koybasi S., Senkal C.E., ElOjeimy S., Liu X., Bielawski J., Day T.A., Gillespie M.B., Sinha D. (2007). Clinical Relevance of Ceramide Metabolism in the Pathogenesis of Human Head and Neck Squamous Cell Carcinoma (HNSCC): Attenuation of C18-Ceramide in HNSCC Tumors Correlates with Lymphovascular Invasion and Nodal Metastasis. Cancer Lett..

[35] Couttas T.A., Kain N., Suchowerska A.K., Quek L.E., Turner N., Fath T., Garner B., Don A.S. (2016). Loss of Ceramide Synthase 2 Activity, Necessary for Myelin Biosynthesis, Precedes Tau Pathology in the Cortical Pathogenesis of Alzheimer’s Disease. Neurobiol. Aging.

[36] Yamamoto M., Sassa T., Kyono Y., Uemura H., Kugo M., Hayashi H., Imai Y., Yamanishi K., Kihara A. (2021). Comprehensive Stratum Corneum Ceramide Profiling Reveals Reduced Acylceramides in Ichthyosis Patient with CERS3 Mutations. J. Dermatol..

[37] Del Rosso J.Q. (2019). Ceramide- and Keratolytic-Containing Body Cleanser and Cream Application in Patients with Psoriasis: Outcomes from a Consumer Usage Study. J. Clin. Anesth. Dermatol..

[38] Hayama T., Hama K., Ozawa T., Fujiwara Y., Nozawa K., Matsuda K., Yokoyama K., Hashiguchi Y., Ochiai H., Misawa T. (2023). Ceramide Synthase CERS4 Gene Downregulation Is Associated with KRAS Mutation in Colorectal Cancer. Sci. Rep..

[39] Lahiri S., Futerman A.H. (2005). LASS5 Is a Bona Fide Dihydroceramide Synthase That Selectively Utilizes Palmitoyl-CoA as Acyl Donor. J. Biol. Chem..

[40] El-Hindi K., Brachtendorf S., Hartel J.C., Oertel S., Birod K., Trautmann S., Thomas D., Ulshöfer T., Weigert A., Utermöhlen O. (2020). Ceramide Synthase 5 Deficiency Aggravates Dextran Sodium Sulfate-Induced Colitis and Colon Carcinogenesis and Impairs T-Cell Activation. Cancers.

[41] Verlekar D., Wei S.-J., Cho H., Yang S., Kang M.H. (2018). Ceramide Synthase-6 Confers Resistance to Chemotherapy by Binding to CD95/Fas in T-Cell Acute Lymphoblastic Leukemia. Cell Death Dis..

[42] Chen H., He B., Ke F. (2022). Ceramide Synthase 6 Mediates Triple-Negative Breast Cancer Response to Chemotherapy Through RhoA- and EGFR-Mediated Signaling Pathways. J. Breast Cancer.

[43] Rodriguez-Cuenca S., Barbarroja N., Vidal-Puig A. (2015). Dihydroceramide Desaturase 1, the Gatekeeper of Cramide Induced Lipotoxicity. Biochim. Biophys. Acta.

[44] Planas-Serra L., Launay N., Goicoechea L., Heron B., Jou C., Juliá-Palacios N., Ruiz M., Fourcade S., Casasnovas C., De La Torre C. (2023). Sphingolipid Desaturase DEGS1 Is Essential for Mitochondria-Associated Membrane Integrity. J. Clin. Investig..

[45] Tzou F.-Y., Hornemann T., Yeh J.-Y., Huang S.-Y. (2023). The Pathophysiological Role of Dihydroceramide Desaturase in the Nervous System. Prog. Lipid Res..

[46] Kumagai K., Yasuda S., Okemoto K., Nishijima M., Kobayashi S., Hanada K. (2005). CERT Mediates Intermembrane Transfer of Various Molecular Species of Ceramides. J. Biol. Chem..

[47] Hanada K. (2010). Intracellular Trafficking of Ceramide by Ceramide Transfer Protein. Proc. Jpn. Acad. Ser. B Phys. Biol. Sci..

[48] Hanada K., Sakai S., Kumagai K. (2022). Natural Ligand-Mimetic and Nonmimetic Inhibitors of the Ceramide Transport Protein CERT. Int. J. Mol. Sci..

[49] Bandet C.L., Hajduch E. (2021). CERT-Dependent Ceramide Transport, A Crucial Process in Cells. J. Diabetes Clin. Res..

[50] Kumagai K., Hanada K. (2019). Structure, Functions and Regulation of CERT, a Lipid-Transfer Protein for the Delivery of Ceramide at the ER–Golgi Membrane Contact Sites. FEBS Lett..

[51] Fitzgerald T.W., Gerety S.S., Jones W.D., Van Kogelenberg M., King D.A., McRae J., Morley K.I., Parthiban V., Al-Turki S., Ambridge K. (2015). Large-Scale Discovery of Novel Genetic Causes of Developmental Disorders. Nature.

[52] Mao C., Obeid L.M. (2008). Ceramidases: Regulators of Cellular Responses Mediated by Ceramide, Sphingosine, and Sphingosine-1-Phosphate. Biochim. Biophys. Acta.

[53] Xiong Z.-J., Huang J., Poda G., Pomès R., Privé G.G. (2016). Structure of Human Acid Sphingomyelinase Reveals the Role of the Saposin Domain in Activating Substrate Hydrolysis. J. Mol. Biol..

[54] Duarte C., Akkaoui J., Yamada C., Ho A., Mao C., Movila A. (2020). Elusive Roles of the Different Ceramidases in Human Health, Pathophysiology, and Tissue Regeneration. Cells.

[55] Kleynerman A., Rybova J., Faber M.L., McKillop W.M., Levade T., Medin J.A. (2023). Acid Ceramidase Deficiency: Bridging Gaps between Clinical Presentation, Mouse Models, and Future Therapeutic Interventions. Biomolecules.

[56] Casasampere M., Camacho L., Cingolani F., Casas J., Egido-Gabás M., Abad J.L., Bedia C., Xu R., Wang K., Canals D. (2015). Activity of Neutral and Alkaline Ceramidases on Fluorogenic N-Acylated Coumarin-Containing Aminodiols. J. Lipid Res..

[57] Gomez-Larrauri A., Das Adhikari U., Aramburu-Nuñez M., Custodia A., Ouro A. (2021). Ceramide Metabolism Enzymes—Therapeutic Targets against Cancer. Medicina.

[58] García-Barros M., Coant N., Kawamori T., Wada M., Snider A.J., Truman J.P., Wu B.X., Furuya H., Clarke C.J., Bialkowska A.B. (2016). Role of Neutral Ceramidase in Colon Cancer. FASEB J..

[59] Sun W., Xu R., Hu W., Jin J., Crellin H.A., Bielawski J., Szulc Z.M., Thiers B.H., Obeid L.M., Mao C. (2008). Upregulation of the Human Alkaline Ceramidase 1 and Acid Ceramidase Mediates Calcium-Induced Differentiation of Epidermal Keratinocytes. J. Investig. Derm..

[60] Sun W., Jin J., Xu R., Hu W., Szulc Z.M., Bielawski J., Obeid L.M., Mao C. (2010). Substrate Specificity, Membrane Topology, and Activity Regulation of Human Alkaline Ceramidase 2 (ACER2). J. Biol. Chem..

[61] Hu W., Xu R., Sun W., Szulc Z.M., Bielawski J., Obeid L.M., Mao C. (2010). Alkaline Ceramidase 3 (ACER3) Hydrolyzes Unsaturated Long-Chain Ceramides, and Its down-Regulation Inhibits Both Cell Proliferation and Apoptosis. J. Biol. Chem..

[62] Vasiliauskaité-Brooks I., Healey R.D., Rochaix P., Saint-Paul J., Sounier R., Grison C., Waltrich-Augusto T., Fortier M., Hoh F., Saied E.M. (2018). Structure of a Human Intramembrane Ceramidase Explains Enzymatic Dysfunction Found in Leukodystrophy. Nat. Commun..

[63] Wang K., Xu R., Snider A., Schrandt J., Li Y., Bialkowska A., Li M., Zhou J., A Hannun Y., Obeid L.M. (2016). Alkaline Ceramidase 3 Deficiency Aggravates Colitis and Colitis-Associated Tumorigenesis in Mice by Hyperactivating the Innate Immune System. Cell Death Dis..

[64] Jiang X.-C., Chiang Y., Jiang X.-C. (2022). Sphingomyelin Synthase Family and Phospholipase Cs. Sphingolipid Metabolism and Metabolic Disease.

[65] Tafesse F.G., Huitema K., Hermansson M., van der Poel S., van den Dikkenberg J., Uphoff A., Somerharju P., Holthuis J.C. (2007). Both Sphingomyelin Synthases SMS1 and SMS2 Are Required for Sphingomyelin Homeostasis and Growth in Human HeLa Cells. J. Biol. Chem..

[66] Futerman A.H., Stieger B., Hubbard A.L., Pagano R.E. (1990). Sphingomyelin Synthesis in Rat Liver Occurs Predominantly at the Cis and Medial Cisternae of the Golgi Apparatus. J. Biol. Chem..

[67] Tafesse F.G., Ternes P., Holthuis J.C.M. (2006). The Multigenic Sphingomyelin Synthase Family. J. Biol. Chem..

[68] Senkal C.E., Salama M.F., Snider A.J., Allopenna J.J., Rana N.A., Koller A., Hannun Y.A., Obeid L.M. (2017). Ceramide Is Metabolized to Acylceramide and Stored in Lipid Droplets. Cell Metab..

[69] Stone S.J., Levin M.C., Zhou P., Han J., Walther T.C., Farese R.V. (2009). The Endoplasmic Reticulum Enzyme DGAT2 Is Found in Mitochondria-Associated Membranes and Has a Mitochondrial Targeting Signal That Promotes Its Association with Mitochondria. J. Biol. Chem..

[70] Wijesinghe D.S., Massiello A., Subramanian P., Szulc Z., Bielawska A., Chalfant C.E. (2005). Substrate Specificity of Human Ceramide Kinase. J. Lipid Res..

[71] Lamour N.F., Stahelin R.V., Wijesinghe D.S., Maceyka M., Wang E., Allegood J.C., Merrill A.H., Cho W., Chalfant C.E. (2007). Ceramide Kinase Uses Ceramide Provided by Ceramide Transport Protein: Localization to Organelles of Eicosanoid Synthesis. J. Lipid Res..

[72] Hannun Y.A., Obeid L.M. (2008). Principles of Bioactive Lipid Signalling: Lessons from Sphingolipids. Nat. Rev. Mol. Cell Biol..

[73] Morell P., Radin N.S. (1969). Synthesis of Cerebroside by Brain from Uridine Diphosphate Galactose and Ceramide Containing Hydroxy Fatty Acid. Biochemistry.

[74] Brenkert A., Radin N.S. (1972). Synthesis of Galactosyl Ceramide and Glucosyl Ceramide by Rat Brain: Assay Procedures and Changes with Age. Brain Res..

[75] Hayashi T., Hayashi E., Fujimoto M., Sprong H., Su T.P. (2012). The Lifetime of UDP-Galactose:Ceramide Galactosyltransferase Is Controlled by a Distinct Endoplasmic Reticulum-Associated Degradation (ERAD) Regulated by Sigma-1 Receptor Chaperones. J. Biol. Chem..

[76] Yamaji T., Hanada K. (2015). Sphingolipid Metabolism and Interorganellar Transport: Localization of Sphingolipid Enzymes and Lipid Transfer Proteins. Traffic.

[77] Sprong H., Kruithof B., Leijendekker R., Slot J.W., van Meer G., van der Sluijs P. (1998). UDP-Galactose:Ceramide Galactosyltransferase Is a Class I Integral Membrane Protein of the Endoplasmic Reticulum. J. Biol. Chem..

[78] Kensinger R.D., Catalone B.J., Krebs F.C., Wigdahl B., Schengrund C.L. (2004). Novel Polysulfated Galactose-Derivatized Dendrimers as Binding Antagonists of Human Immunodeficiency Virus Type 1 Infection. Antimicrob. Agents Chemother..

[79] Svennerholm L., Vanier M., Månsson J. (1980). Krabbe Disease: A Galactosylsphingosine (Psychosine) Lipidosis. J. Lipid Res..

[80] Chen S., Wang Y., Kong L., Ji Y., Cui J., Shen W. (2023). Role of UDP-Glucose Ceramide Glucosyltransferase in Venous Malformation. Front. Cell Dev. Biol..

[81] Batta G., Soltész L., Kovács T., Bozó T., Mészár Z., Kellermayer M., Szöllősi J., Nagy P. (2018). Alterations in the Properties of the Cell Membrane Due to Glycosphingolipid Accumulation in a Model of Gaucher Disease. Sci. Rep..

[82] Mullen T.D., Hannun Y.A., Obeid L.M. (2012). Ceramide Synthases at the Centre of Sphingolipid Metabolism and Biology. Biochem. J..

[83] Maula T., Artetxe I., Grandell P.M., Slotte J.P. (2012). Importance of the Sphingoid Base Length for the Membrane Properties of Ceramides. Biophys. J..

[84] Lunghi G., Fazzari M., Di Biase E., Mauri L., Chiricozzi E., Sonnino S. (2021). The Structure of Gangliosides Hides a Code for Determining Neuronal Functions. FEBS Open Bio.

[85] Pinto S.N., Silva L.C., Futerman A.H., Prieto M. (2011). Effect of Ceramide Structure on Membrane Biophysical Properties: The Role of Acyl Chain Length and Unsaturation. Biochim. Biophys. Acta (BBA)—Biomembr..

[86] Knothe G., Dunn R.O. (2009). A Comprehensive Evaluation of the Melting Points of Fatty Acids and Esters Determined by Differential Scanning Calorimetry. J. Am. Oil Chem. Soc..

[87] Bode L., Beermann C., Mank M., Kohn G., Boehm G. (2004). Human and Bovine Milk Gangliosides Differ in Their Fatty Acid Composition. J. Nutr..

[88] Liyanage O.T., Xia C., Ringler S., Stahl B., Costello C.E. (2023). Defining the Ceramide Composition of Bovine and Human Milk Gangliosides by Direct Infusion ESI-CID Tandem Mass Spectrometry of Native and Permethylated Molecular Species. Anal. Chem..

[89] Eom M., Yoon H., Jhon G. (1996). Changes of Gangliosides and Sialic Acid in Human Milk during Lactation. Exp. Mol. Med..

[90] D’Angelo G., Uemura T., Chuang C.C., Polishchuk E., Santoro M., Ohvo-Rekilä H., Sato T., Di Tullio G., Varriale A., D’auria S. (2013). Vesicular and Non-Vesicular Transport Feed Distinct Glycosylation Pathways in the Golgi. Nature.

[91] Chung L., Liu D., Liu X., Qi Y. (2021). Ceramide Transfer Protein (CERT): An Overlooked Molecular Player in Cancer. Int. J. Mol. Sci..

[92] Nakashima K., Hirahara Y., Koike T., Tanaka S., Gamo K., Oe S., Hayashi S., Seki-Omura R., Nakano Y., Ohe C. (2022). Sulfatide with Ceramide Composed of Phytosphingosine (T18:0) and 2-Hydroxy FAs in Renal Intercalated Cells. J. Lipid Res..

[93] Hama H. (2010). Fatty Acid 2-Hydroxylation in Mammalian Sphingolipid Biology. Biochim. Biophys. Acta.

[94] Mishra S.K., Gao Y.G., Zou X., Stephenson D.J., Malinina L., Hinchcliffe E.H., Chalfant C.E., Brown R.E. (2020). Emerging Roles for Human Glycolipid Transfer Protein Superfamily Members in the Regulation of Autophagy, Inflammation, and Cell Death. Prog. Lipid Res..

[95] Backman A.P.E., Halin J., Nurmi H., Möuts A., Kjellberg M.A., Mattjus P. (2018). Glucosylceramide Acyl Chain Length Is Sensed by the Glycolipid Transfer Protein. PLoS ONE.

[96] Lauria I., van Üüm J., Mjumjunov-Crncevic E., Walrafen D., Spitta L., Thiele C., Lang T. (2013). GLTP Mediated Non-Vesicular GM1 Transport between Native Membranes. PLoS ONE.

[97] Nurmi H., Backman A.P.E., Halin J., Lönnfors M., Blom T., Roos-Mattjus P., Mattjus P. (2023). Glycolipid Transfer Protein Knockout Disrupts Vesicle Trafficking to the Plasma Membrane. J. Biol. Chem..

[98] Malinina Lucy L., Simanshu D.K., Zhai X., Samygina V.R., Kamlekar R., Kenoth R., Ochoa-Lizarralde B., Malakhova M.L., Molotkovsky J.G., Patel D.J. (2015). Sphingolipid Transfer Proteins Defined by the GLTP-Fold. Q. Rev. Biophys..

[99] Mattjus P. (2016). Specificity of the Mammalian Glycolipid Transfer Proteins. Chem. Phys. Lipids.

[100] Martínez-Palomo A., Bourne G.H., Danielli J.F., Jeon K.W. (1970). The Surface Coats of Animal Cells11Part of the Personal Work Mentioned in This Review Was Performed at the Institut de Recherches Scientifiques Sur Le Cancer, Villejuif, France. International Review of Cytology.

[101] Vyas K.A., Patel H.V., Vyas A.A., Schnaar R.L. (2001). Segregation of Gangliosides GM1 and GD3 on Cell Membranes, Isolated Membrane Rafts, and Defined Supported Lipid Monolayers. Biol. Chem..

[102] Itokazu Y., Yu R.K. (2023). Ganglioside Microdomains on Cellular and Intracellular Membranes Regulate Neuronal Cell Fate Determination. Adv. Neurobiol..

[103] Chowdhury S., Ledeen R. (2022). The Key Role of GM1 Ganglioside in Parkinson’s Disease. Biomolecules.

[104] Bos P.D., Zhang X.H., Nadal C., Shu W., Gomis R.R., Nguyen D.X., Minn A.J., van de Vijver M.J., Gerald W.L., Foekens J.A. (2009). Genes That Mediate Breast Cancer Metastasis to the Brain. Nature.

[105] Yoshida H., Koodie L., Jacobsen K., Hanzawa K., Miyamoto Y., Yamamoto M. (2020). B4GALNT1 Induces Angiogenesis, Anchorage Independence Growth and Motility, and Promotes Tumorigenesis in Melanoma by Induction of Ganglioside GM2/GD2. Sci. Rep..

[106] Iwabuchi K., Nakayama H., Oizumi A., Suga Y., Ogawa H., Takamori K. (2015). Role of Ceramide from Glycosphingolipids and Its Metabolites in Immunological and Inflammatory Responses in Humans. Mediat. Inflamm..

[107] Grayson G., Ladisch S. (1992). Immunosuppression by human gangliosides: II. Carbohydrate structure and inhibition of human NK activity. Cell. Immun..

[108] Svennerholm L. (1980). Ganglioside Designation. Adv. Exp. Med. Biol..

[109] Blix F.G., Gottschalk A., Klenk E. (1957). Proposed Nomenclature in the Field of Neuraminic and Sialic Acids. Nature.

[110] Svennerholm L. (1980). Gangliosides and Synaptic Transmission. Structure and Function of Gangliosides.

[111] Caughlin S., Maheshwari S., Weishaupt N., Yeung K.K.-C., Cechetto D.F., Whitehead S.N. (2017). Age-Dependent and Regional Heterogeneity in the Long-Chain Base of A-Series Gangliosides Observed in the Rat Brain Using MALDI Imaging. Sci. Rep..

[112] Kawai H., Allende M.L., Wada R., Kono M., Sango K., Deng C., Miyakawa T., Crawley J.N., Werth N., Bierfreund U. (2001). Mice Expressing Only Monosialoganglioside GM3 Exhibit Lethal Audiogenic Seizures. J. Biol. Chem..

[113] Simpson M.A., Cross H., Proukakis C., Priestman D.A., Neville D.C., Reinkensmeier G., Wang H., Wiznitzer M., Gurtz K., Verganelaki A. (2004). Infantile-Onset Symptomatic Epilepsy Syndrome Caused by a Homozygous Loss-of-Function Mutation of GM3 Synthase. Nat. Genet..

[114] Breiden B., Sandhoff K., Sonnino S., Prinetti A. (2018). Ganglioside Metabolism and Its Inherited Diseases. Gangliosides: Methods and Protocols.

[115] Bremer E.G., Schlessinger J., Hakomori S.-I. (1986). Ganglioside-Mediated Modulation of Cell Growth. Specific Effects of GM3 on Tyrosine Phosphorylation of the Epidermal Growth Factor Receptor. J. Biol. Chem..

[116] Hamamura K., Furukawa K., Hayashi T., Hattori T., Nakano J., Nakashima H., Okuda T., Mizutani H., Hattori H., Ueda M. (2005). Ganglioside GD3 Promotes Cell Growth and Invasion through p130Cas and Paxillin in Malignant Melanoma Cells. Proc. Natl. Acad. Sci. USA.

[117] McGonigal R., Willison H.J. (2022). The Role of Gangliosides in the Organisation of the Node of Ranvier Examined in Glycosyltransferase Transgenic Mice. J. Anat..

[118] Sachs B. (1887). On Arrested Cerebral Development, With Special Reference to Its Cortical Pathology. J. Nerv. Ment. Dis..

[119] Matsuzaki K., Kato K., Yanagisawa K. (2010). Aβ Polymerization through Interaction with Membrane Gangliosides. Biochim. Biophys. Acta.

[120] Shin M.-K., Choi M.-S., Chae H.-J., Kim J.-W., Kim H.-G., Kim K.-L. (2019). Ganglioside GQ1b Ameliorates Cognitive Impairments in an Alzheimer’s Disease Mouse Model, and Causes Reduction of Amyloid Precursor Protein. Sci. Rep..

[121] Chung T.W., Kim S.J., Choi H.J., Kim K.J., Kim M.J., Kim S.H., Lee H.-J., Ko J.-H., Lee Y.-C., Suzuki A. (2009). Ganglioside GM3 Inhibits VEGF/VEGFR-2-Mediated Angiogenesis: Direct Interaction of GM3 with VEGFR-2. Glycobiology.

[122] Varki A., Cummings R.D., Aebi M., Packer N.H., Seeberger P.H., Esko J.D., Stanley P., Hart G., Darvill A., Kinoshita T. (2015). Symbol Nomenclature for Graphical Representations of Glycans. Glycobiology.

[123] Nishie T., Hikimochi Y., Zama K., Fukusumi Y., Ito M., Yokoyama H., Naruse C., Ito M., Asano M. (2010). Β4-Galactosyltransferase-5 Is a Lactosylceramide Synthase Essential for Mouse Extra-Embryonic Development. Glycobiology.

[124] Tokuda N., Numata S., Li X., Nomura T., Takizawa M., Kondo Y., Yamashita Y., Hashimoto N., Kiyono T., Urano T. (2013). β4GalT6 Is Involved in the Synthesis of Lactosylceramide with Less Intensity than β4GalT5. Glycobiology.

[125] Lipina C., Hundal H.S. (2015). Ganglioside GM3 as a Gatekeeper of Obesity-Associated Insulin Resistance: Evidence and Mechanisms. FEBS Lett..

[126] Kabayama K., Sato T., Kitamura F., Uemura S., Kang B.W., Igarashi Y., Inokuchi J. (2005). TNFα-Induced Insulin Resistance in Adipocytes as a Membrane Microdomain Disorder: Involvement of Ganglioside GM3. Glycobiology.

[127] Inokuchi J., Inamori K., Kabayama K., Nagafuku M., Uemura S., Go S., Suzuki A., Ohno I., Kanoh H., Shishido F., Schnaar R.L., Lopez P.H.H. (2018). Chapter Five—Biology of GM3 Ganglioside. Progress in Molecular Biology and Translational Science.

[128] Wang X., Yu H., Gao R., Liu M., Xie W. (2023). A Comprehensive Review of the Family of Very-Long-Chain Fatty Acid Elongases: Structure, Function, and Implications in Physiology and Pathology. Eur. J. Med. Res..

[129] Inokuchi J.I., Kanoh H. (2022). Pathophysiological Significance of GM3 Ganglioside Molecular Species with a Particular Attention to the Metabolic Syndrome Focusing on Toll-Like Receptor 4 Binding. Front. Mol. Biosci..

[130] Oishi Y., Spann N.J., Link V.M., Muse E.D., Strid T., Edillor C., Kolar M.J., Matsuzaka T., Hayakawa S., Tao J. (2017). SREBP1 Contributes to Resolution of Pro-Inflammatory TLR4 Signaling by Reprogramming Fatty Acid Metabolism. Cell Metab..

[131] Pinto S.N., Laviad E.L., Stiban J., Kelly S.L., Merrill A.H., Prieto M., Futerman A.H., Silva L.C. (2014). The Sphingoid Bases of Sphingolipids, Including Ceramides, Can Vary in Length from 12 to >20 Carbons. J. Lipid Res..

[132] Ogawa-Goto K., Funamoto N., Abe T., Nagashima K. (1990). Different Ceramide Compositions of Gangliosides between Human Motor and Sensory Nerves. J. Neurochem..

[133] Ma L., Fong B.Y., MacGibbon A.K.H., Norris G. (2020). Qualitative and Quantitative Study of Glycosphingolipids in Human Milk and Bovine Milk Using High Performance Liquid Chromatography-Data-Dependent Acquisition-Mass Spectrometry. Molecules.

[134] Chiricozzi E., Ciampa M.G., Brasile G., Compostella F., Prinetti A., Nakayama H., Ekyalongo R.C., Iwabuchi K., Sonnino S., Mauri L. (2015). Direct Interaction, Instrumental for Signaling Processes, between LacCer and Lyn in the Lipid Rafts of Neutrophil-like Cells. J. Lipid Res..

[135] Chatterjee S., Balram A., Li W. (2021). Convergence: Lactosylceramide-Centric Signaling Pathways Induce Inflammation, Oxidative Stress, and Other Phenotypic Outcomes. Int. J. Mol. Sci..

[136] Park E.J., Suh M., Clandinin M.T. (2005). Dietary Ganglioside and Long-Chain Polyunsaturated Fatty Acids Increase Ganglioside GD3 Content and Alter the Phospholipid Profile in Neonatal Rat Retina. Investig. Opthalmol. Vis. Sci..

[137] Kanoh H., Nitta T., Go S., Inamori K., Veillon L., Nihei W., Fujii M., Kabayama K., Shimoyama A., Fukase K. (2020). Homeostatic and Pathogenic Roles of GM 3 Ganglioside Molecular Species in TLR 4 Signaling in Obesity. EMBO J..

[138] Veillon L., Go S., Matsuyama W., Suzuki A., Nagasaki M., Yatomi Y., Inokuchi J.-I. (2015). Identification of Ganglioside GM3 Molecular Species in Human Serum Associated with Risk Factors of Metabolic Syndrome. PLoS ONE.

[139] Colsch B., Jackson S.N., Dutta S., Woods A.S. (2011). Molecular Microscopy of Brain Gangliosides: Illustrating Their Distribution in Hippocampal Cell Layers. ACS Chem. Neurosci..

[140] Nakatani Y., Yanagisawa M., Suzuki Y., Yu R.K. (2010). Characterization of GD3 Ganglioside as a Novel Biomarker of Mouse Neural Stem Cells. Glycobiology.

[141] Matarrese P., Garofalo T., Manganelli V., Gambardella L., Marconi M., Grasso M., Tinari A., Misasi R., Malorni W., Sorice M. (2014). Evidence for the Involvement of GD3 Ganglioside in Autophagosome Formation and Maturation. Autophagy.

[142] Ando S., Yu R.K. (1984). Fatty Acid and Long-Chain Base Composition of Gangliosides Isolated from Adult Human Brain. J. Neurosci. Res..

[143] Schengrund C. (2023). The Ying and Yang of Ganglioside Function in Cancer. Cancers.

[144] Vajn K., Viljetic B., Degmecic V., Schnaar R.L., Heffer M. (2013). Differential Distribution of Major Brain Gangliosides in the Adult Mouse Central Nervous System. PLoS ONE.

[145] Aureli M., Mauri L., Ciampa M.G., Prinetti A., Toffano G., Secchieri C., Sonnino S. (2016). GM1 Ganglioside: Past Studies and Future Potential. Mol. Neurobiol..

[146] Arumugam S., Schmieder S., Pezeshkian W., Becken U., Wunder C., Chinnapen D., Ipsen J.H., Kenworthy A.K., Lencer W., Mayor S. (2021). Ceramide Structure Dictates Glycosphingolipid Nanodomain Assembly and Function. Nat. Commun..

[147] Schmieder S.S., Tatituri R., Anderson M., Kelly K., Lencer W.I. (2022). Structural Basis for Acyl Chain Control over Glycosphingolipid Sorting and Vesicular Trafficking. Cell Rep..

[148] Chowdhury S., Wu G., Lu Z.-H., Kumar R., Ledeen R. (2023). Age-Related Decline in Gangliosides GM1 and GD1a in Non-CNS Tissues of Normal Mice: Implications for Peripheral Symptoms of Parkinson’s Disease. Biomedicines.

[149] Mitsuda T., Furukawa K., Fukumoto S., Miyazaki H., Urano T., Furukawa K. (2002). Overexpression of Ganglioside GM1 Results in the Dispersion of Platelet-Derived Growth Factor Receptor from Glycolipid-Enriched Microdomains and in the Suppression of Cell Growth Signals. J. Biol. Chem..

[150] Taki T. (2012). An Approach to Glycobiology from Glycolipidomics: Ganglioside Molecular Scanning in the Brains of Patients with Alzheimer’s Disease by TLC-Blot/Matrix Assisted Laser Desorption/Ionization-Time of Flight MS. Biol. Pharm. Bull..

[151] Sarbu M., Ica R., Zamfir A.D. (2022). Gangliosides as Biomarkers of Human Brain Diseases: Trends in Discovery and Characterization by High-Performance Mass Spectrometry. Int. J. Mol. Sci..

[152] Yanagisawa K. (2015). GM1 Ganglioside and Alzheimer’s Disease. Glycoconj. J..

[153] Tagliapietra M., Zanusso G., Ferrari S., Orlandi R., Bertolasi L., Cavallaro T., Monaco S. (2020). Myelin Uncompaction and Axo-glial Detachment in Chronic Ataxic Neuropathy with Monospecific IgM Antibody to Ganglioside GD1b. J. Peripher. Nerv. Syst..

[154] Lim H., Lee J., You B., Oh J.H., Mok H.J., Kim Y.S., Yoon B.-E., Kim B.G., Back S.K., Park J.-S. (2020). GT1b Functions as a Novel Endogenous Agonist of Toll-like Receptor 2 Inducing Neuropathic Pain. EMBO J..

[155] Ica R., Mlinac-Jerkovic K., Ilic K., Sajko T., Munteanu C.V.A., Zamfir A.D., Kalanj-Bognar S. (2022). Gangliosidome of a Human Hippocampus in Temporal Lobe Epilepsy Resolved by High-Resolution Tandem Mass Spectrometry. Molecules.

[156] Svennerholm L., Bråne G., Karlsson I., Lekman A., Ramström I., Wikkelsö C. (2002). Alzheimer Disease–Effect of Continuous Intracerebroventricular Treatment with GM1 Ganglioside and a Systematic Activation Programme. Dement. Geriatr. Cogn. Disord..

[157] Schneider J.S. (2018). Altered Expression of Genes Involved in Ganglioside Biosynthesis in Substantia Nigra Neurons in Parkinson’s Disease. PLoS ONE.

[158] Magistretti P.J., Geisler F.H., Schneider J.S., Li P.A., Fiumelli H., Sipione S. (2019). Gangliosides: Treatment Avenues in Neurodegenerative Disease. Front. Neurol..

[159] Groux-Degroote S., Delannoy P. (2021). Cancer-Associated Glycosphingolipids as Tumor Markers and Targets for Cancer Immunotherapy. Int. J. Mol. Sci..

[160] Valentino L., Moss T., Olson E., Wang H.-J., Elashoff R., Ladisch S. (1990). Shed Tumor Gangliosides and Progression of Human Neuroblastoma. Blood.

[161] van der Haar Àvila I., Windhouwer B., van Vliet S.J. (2023). Current State-of-the-Art on Ganglioside-Mediated Immune Modulation in the Tumor Microenvironment. Cancer Metastasis Rev..

[162] Balis F.M., Busch C.M., Desai A.V., Hibbitts E., Naranjo A., Bagatell R., Irwin M., Fox E. (2020). The Ganglioside GD2 as a Circulating Tumor Biomarker for Neuroblastoma. Pediatr. Blood Cancer.

[163] Nazha B., Inal C., Owonikoko T.K. (2020). Disialoganglioside GD2 Expression in Solid Tumors and Role as a Target for Cancer Therapy. Front. Oncol..

[164] Voeller J., Sondel P.M. (2019). Advances in Anti-GD2 Immunotherapy for Treatment of High-Risk Neuroblastoma. J. Pediatr. Hematol. Oncol..

[165] Cohn S.L., Pearson A.D.J., London W.B., Monclair T., Ambros P.F., Brodeur G.M., Faldum A., Hero B., Iehara T., Machin D. (2009). The International Neuroblastoma Risk Group (INRG) Classification System: An INRG Task Force Report. J. Clin. Oncol..

[166] Desai A.V., Gilman A.L., Ozkaynak M.F., Naranjo A., London W.B., Tenney S.C., Diccianni M., Hank J.A., Parisi M.T., Shulkin B.L. (2022). Outcomes Following GD2-Directed Postconsolidation Therapy for Neuroblastoma After Cessation of Random Assignment on ANBL0032: A Report from the Children’s Oncology Group. J. Clin. Oncol..

[167] Zhao Z., Nelson A.R., Betsholtz C., Zlokovic B.V. (2015). Establishment and Dysfunction of the Blood-Brain Barrier. Cell.

[168] Gobburi A.L.P., Kipruto E.W., Inman D.M., Anderson D.J. (2021). A New LC-MS/MS Technique for Separation of Gangliosides Using a Phenyl-Hexyl Column: Systematic Separation According to Sialic Acid Class and Ceramide Subclass. J. Liq. Chromatogr. Relat. Technol..

[169] Biricioiu M.R., Sarbu M., Ica R., Vukelić Ž., Kalanj-Bognar S., Zamfir A.D. (2024). Advances in Mass Spectrometry of Gangliosides Expressed in Brain Cancers. Int. J. Mol. Sci..

[170] Hohenwallner K., Troppmair N., Panzenboeck L., Kasper C., El Abiead Y., Koellensperger G., Lamp L.M., Hartler J., Egger D., Rampler E. (2022). Decoding Distinct Ganglioside Patterns of Native and Differentiated Mesenchymal Stem Cells by a Novel Glycolipidomics Profiling Strategy. JACS Au.

[171] Sonnino S., Mauri L., Chigorno V., Prinetti A. (2007). Gangliosides as Components of Lipid Membrane Domains. Glycobiology.

[172] Jin X., Cheng H., Chen X., Cao X., Xiao C., Ding F., Qu H., Wang P.G., Feng Y., Yang G.-Y. (2024). A Modular Chemoenzymatic Cascade Strategy for the Structure-Customized Assembly of Ganglioside Analogs. Commun. Chem..

